# Gastrointestinal parasites of harbour seal (*Phoca vitulina* L.) in Danish marine waters: Prevalence, abundance, intensity and reproductive potential

**DOI:** 10.1016/j.ijppaw.2025.101066

**Published:** 2025-04-01

**Authors:** Kaan Kumas, Carlota Marola Fernandez Gonzalez, Per Walter Kania, Kurt Buchmann

**Affiliations:** Laboratory of Aquatic Pathobiology, Department of Veterinary and Animal Sciences, Faculty of Health and Medical Sciences, University of Copenhagen, Frederiksberg C, Denmark

**Keywords:** *Anisakis*, *Phocanema*, *Contracaecum*, *Dibothriocephalus*, *Corynosoma*, Infection risk

## Abstract

Populations of harbour seal (*Phoca vitulina*) in Danish marine areas have increased markedly during recent decades, but their associated parasite populations have remained unknown. In February 2024 we collected 13 harbour seals from the western part of Limfjorden (Denmark), a marine area connecting the North Sea with the Kattegat Sea. The animals were subjected to a parasitological examination (including morphological and molecular methods), which confirmed that this seal species acts as a definitive hosts for the gastrointestinal nematodes *Contracaecum osculatum s.s.*, *Anisakis simplex*, *Phocanema decipiens s.s.* and *P. krabbei*; all nematodes were at prevalences of 100 %. The seals also harboured the intestinal acanthocephalan *Corynosoma strumosum* (100 % prevalence) and the cestode *Dibothriocephalus schistochilus* (7.7 % prevalence). The nematode intensities ranged from 4 to 1790 individuals per host, whereas acanthocephalans occurred in lower numbers (range 4–222 per host) and a single seal was infected with three cestodes. The reproductive potential of the nematodes was evaluated by counting mature eggs in female worms, which indicated that each of the female worms could release between 7202 and 72,810 eggs per day. Infection intensities revealed that a single harbour seal on average releases more than 3 × 10^5^ eggs per day. In more heavily infected seals, the potential release of anisakid eggs to the environment exceeds 2.4 × 10^6^ eggs per day, each of which, after hatching and invasion of the first paratenic host, represents a potential infection risk for fish and humans. Some of the anisakid parasites are potentially infective to human consumers, and we discuss the potential spread of infection to fish and humans at different infection levels and seal population sizes.

## Highlights

• Nematodes, acanthocephalans and cestodes occur in harbour seal from Danish marine waters.
• *Anisakis simplex, Contracaecum* osculatum, *Phocanema decipiens* and *P. krabbei* infect seal stomach.
• Adult acanthocephalans *Corynosoma* and cestodes *Dibothriocephalus* occur in the seal intestine.
• Harbour seal supports full sexual development of *Anisakis simplex.*
• Seal parasites produce a high infection pressure on local fish stocks.

## Graphical abstract

**Figure:**
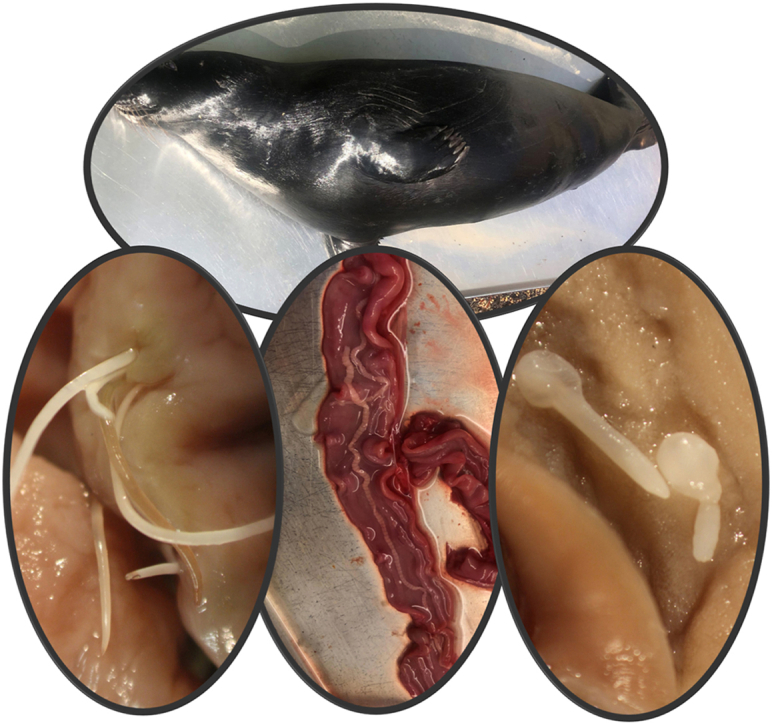

## 1 Introduction

Harbour seal (*Phoca vitulina*) populations occur along the eastern and western coasts of the North Atlantic and North Pacific Oceans from temperate to sub-Arctic waters. They are represented by four recognized marine subspecies comprising 1) the eastern North Atlantic harbour seal *P. v. vitulina* (Linnaeus, 1758), 2) the western North Atlantic harbour seal *P. v. concolor* (DeKay, 1842), 3) the eastern North Pacific harbour seal *P. v. richardii* (Grabda, 1976) and 4) the western North Pacific harbour seal *P. v. stejnegeri* (Allende et al., 2024). The distribution of the eastern North Atlantic harbour seal follows the coasts of United Kingdom, Ireland, France, Belgium, Netherlands, Germany, Denmark, Iceland, Sweden, the Russian Federation, and Poland (Bjørge et al., 2010). Over the last 20 years, the harbour seal population has increased markedly in the eastern North Sea (Blanchet et al., 2021); inner Danish waters such as the Limfjorden, Skagerrak, and Kattegat (Olsen et al., 2010); and the Baltic Sea (Harding et al., 2007, 2023; Härkönen and Isakson, 2010). Many parasites of seals have complex life cycles, which involve intermediate or paratenic hosts (invertebrates and fish). Anisakid nematodes use marine mammals as definitive hosts and crustaceans and fish as paratenic hosts (Køie and Fagerholm, 1995; Køie et al., 1995; McClelland, 2002). The nematodes are considered zoonotic (EFSA BIOHAZ Panel, I et al., 2024), and their occurrence may therefore have implications for public health.

The zoonotic potential of the nematode *Phocanema decipiens sensu stricto* (*s.s.*) has been clearly indicated over the last 30 years in numerous reports of human infections (Pinel et al., 1996; Koh et al., 1999; Yu et al., 2001; Na et al., 2013; Cavallero et al., 2016; Brunet et al., 2017; Nordholm et al., 2020; Mejer et al., 2022). Human infections with *Anisakis simplex* are also common (Andreassen and Jørring, 1970; Skirnisson, 2022; Murata et al., 2021). Fewer reports exist regarding human infections with larvae of *Contracaecum osculatum*, but evidence strongly suggests a similar risk associated with this species (Schaum and Müller, 1967; Shamsi and Butcher, 2011; Nagasawa, 2012; Strøm et al., 2015). Even fewer reports have indicated that larvae of seal cestodes (Schaeffner et al., 2018) and seal acanthocephalans (Buchmann and Karami, 2024) may reach and infect human consumers.

This study emphasizes the public health relevance of these helminth parasites occurring in local seal populations. Due to the fact that larvae of these parasites occur in various fish species (as paratenic hosts), it is also worthwhile to consider their importance for fish health when performing seal parasite surveys. Indirect effects on humans and fish were rarely in focus in previous studies describing the endo-parasitic fauna of harbour seals in the North Sea and the North Atlantic (Borgsteede et al., 1991; Aspholm et al., 1995; Marcogliese et al., 1996; Ólafsdóttir and Hauksson, 1998; Lehnert et al., 2007; Lakemeyer et al., 2020). Gastrointestinal parasites in harbour seal found dead along the inner Danish coastlines due to a canine distemper virus epidemic in 1988 were reported by Lunneryd (1991), but we have no updated knowledge on the occurrence of gastrointestinal parasites in harbour seals in Danish marine areas. In order to assess the potential spread of seal-associated parasites to local fish stocks, we need to obtain precise data on infection level and fecundity of the anisakid worms in seals. In this study we thus aim to describe gastrointestinal helminth species, their distribution and infection levels in harbour seals captured in a specific marine location (Limfjorden, Denmark). Our main goal was to estimate the number of nematode eggs released from the seals and secondarily indicate the infection risks for fish and humans.

## 2 Materials and methods

### 2.1 Seal sampling

In February 2024 we recovered 13 harbour seals (*Phoca vitulina*), which were found dead, as they were accidentally trapped in fishing gear of commercial fishermen from the Limfjorden (Nissum Bredning 56°36′55.1″N 8°23′59.6″E), a marine area connecting the North Sea with the Kattegat Sea, Denmark. When brought ashore (6–8 h post-capture) by local fishermen the seals were immediately placed on ice for cooling in 1 m^3^ containers and transported 4 h by car to the University of Copenhagen, Laboratory of Aquatic Pathobiology, where they were frozen (−20 °C) until parasitological accidentally were initiated 1–3 months later.

### 2.2 Necropsy and parasite recovery

Following thawing we recorded the total length and weight of each seal. Necropsy was conducted according to general procedures for mammals (Jensen, 2011). The heart, liver, lungs, and kidneys of each seal were not included in the present study. The gastrointestinal tract was divided into sections, which were subjected to separate examinations for parasites. Gastrointestinal parasites were isolated using forceps under a dissection microscope (Leica MZ125, Germany), rinsed in physiological saline and finally preserved in 96 % ethanol at 4 °C.

### 2.3 Morphological identification

Selected nematodes were sectioned into three parts (anterior, middle and posterior). We used the anterior and posterior ends for morphological analysis (light microscopy), whereas the middle part was placed back in ethanol (96 %) and used for molecular identification. Acanthocephalans were mounted as whole specimens, whereas the anterior attachment organ (scolex) of the cestode was cut by a scalpel and removed for processing. Worm parts sampled for microscopy were cleared with lactic acid for 1 h and mounted on microscope slides in glycerine-gelatine (Buchmann, 2007). The mounted worms (for nematodes the anterior and posterior ends) were examined under a phase-contrast microscope (Leica DM5000B, Germany) at different magnifications (100× and 400X). Morphological identification of each specimen to the genus level was conducted using microscopic examination of the anterior (structure and location of labiae, ventriculus and excretory pore) and posterior end structures (Krabbe, 1878; Val’ter et al., 1982). The acanthocephalans and cestodes were mounted on microscope slides using glycerine-gelatine and morphological analysis (light microscopy) was carried out according to Leidenberger et al. (2019) and Schaeffner et al. (2018), respectively. With regard to identification of acanthocephalans the genus and species characteristics were shape and size of proboscis hooks and the distribution pattern of tegumental spines on the body (Leidenberger et al., 2019). For species determination, male worm body size and morphology (testes and cement glands) was used as well.

The assignment to genus to the isolated cestode we applied the characteristics of the scolex (presence or absence of sclerites) and the number, shape and size of attachment organs (bothria) (Schaeffner et al., 2018). Segments of the acanthocephalans and cestodes were processed similarly to those of the nematodes for molecular identification.

### 2.4 Molecular identification (PCR, sequencing and phylogenetic analysis)

Genomic DNA of the parasites was purified using the QIAamp DNA Mini Kit (cat.no. 61306, Qiagen, Denmark) according to the manufacturer's instructions except that 50 μl elution buffer was used. PCRs were conducted in a BioRad T100 Thermal Cycler (cat.no. 1861096, Bio-Rad Laboratories, Denmark) using reaction volumes of 60 μl with 1 mM forward primer, 1 mM reverse primer (both synthesized at Tag Copenhagen, Denmark), 1 mM dNTP mix (dNTP Blend, cat.no. 10085714, Fisher Scientific, Denmark), 0.6 μl DNA Polymerase, 6 μl 10x Reaction buffer, 1.8 μl 50 mM MgCl2 (BIOTAQ DNA Polymerase, cat.no BIO-21060, Saveen & Werner ApS, Denmark). PCR conditions were pre-denaturation at 95 °C for 5 min, followed by 40 amplification cycles of denaturation at 95 °C for 30 s, annealing at primer set–specific temperatures, elongation at 72 °C for an amount of time dependent on expected product length and finally post-elongation at 72 °C for 7 min. Primer specific conditions, primer combinations and other primer information are listed in Supplementary file S1 in the sheet named “PCR primers etc.” Two primer combinations used touchdown procedures (Td) as outlined in S1. The PCR products were visualised using 1.5 % agarose gel-electrophoresis. We selected a total of 145 parasite specimens for molecular analysis. These comprised 29 *Anisakis simplex*, 51 *Contracaecum osculatum*, 17 *Phocanema decipiens*, 21 *Phocanema krabbei*, 24 *Corynosoma strumosum*, and 3 *Dibothriocephalus schistochilos* specimens. These DNA isolated from these worms were subjected to PCR (in most cases targeting both the genomic ITS region and the mitochondrial *cox2* gene) and the PCR products sequenced.

PCR products were purified using the Illustra™ GFX™ PCR DNA and Gel Band Purification Kit (cat.no. 28-9034-71, VWR International A/S, Denmark), sequenced at Macrogen Europe, Netherlands, and analysed with CLC – Main Workbench v20.0.4 (QIAGEN, Denmark). If present, overlapping PCR products from the same sample were assembled (e.g. PCR products from ERIB1 vs ERIB10 and PCR products from PDG_18S_F5 vs NC2). Multiple alignments and phylogenetic analyses were performed using the same software. Different primer sets and combinations were applied for the three major groups (nematodes, acanthocephalans, cestodes) (Suppl. File S1). As the various primer combinations resulted in products of various lengths, the analysis were restricted to part of the sequences between the forward primers 211F/211F_alt (both having identical binding site) and the reverse primer 210R in the case of the mitochondrial gene cytochrome oxidase II (*cox2*). For full-length ITS (internal transcribed spacer), the 18S 3′ ends and the 28S 5’ ends were included in constructing the alignment, but they were omitted in further phylogenetic analysis of ITS1 – 5.8S – ITS2. Alignments were constructed using Clustal W, and then used for model testing. The CLC – Main Workbench used four different statistical analyses (Hierarchical likelihood ratio test (hLRT), Bayesian information criterion (BIC), Minimum theoretical information criterion (AIC), and Minimum corrected theoretical information criterion (AICc)) to test for five substitution models (Jukes and Cantor, 1969; Kimura, 1980; Felsenstein, 1981; Hasegawa et al., 1985) and General Time Reversible (GTR) (Yang, 1994). The best evolutionary model was determined to be GTR + G + T for both mitochondrial and nuclear sequences. In the CLC software, phylogenetic trees were inferred using the maximum likelihood (ML) method (1000 bootstrap replicates) using an initial tree constructed by UPGMA (100 bootstrap replicates). Only the nematodes were subjected to phylogenetic analysis as a certain degree of diversity was apparent in this group.

### 2.5 Enumerating mature eggs in female nematodes

We selected adult female nematodes, which were assigned to the genera *Anisakis*, *Phocanema* and *Contracaecum* by morphological criteria (see 2.3), and isolated eggs from their uteri following digestion in a volume of 200 μL tissue lysis buffer (180 μL ATL with 20 μL proteinase K, QIAGEN Gmbh, Germany) for at least 24 h at 56 °C. The supernatant was then removed for DNA isolation and molecular identification (PCR and sequencing) of the female worm delivering the eggs. Mature nematode eggs were resistant to this digestion process, which allowed us to isolate them for microscopy and counting. The isolated eggs had a sticky coating on the surface and had to be pretreated (72 h at 56 °C with 1 % Triton X-100 in 1 mL RNAse free water (Calbiochem, MA, USA) to remove this coating. They were then transferred to wells with water in a 96-well plate for counting (20 μL suspension per well, F-bottom, 655-180, Greiner Bio-One, Germany). We used an inverted microscope (magnification 50× and 100X) (Olympus CK-40-F200, Japan) and enumerated all nematode eggs (not subsamples) from the individual female parasites. Subsamples were taken randomly for measurement of parasite egg dimensions (length, width).

### 2.6 Statistics

Parasitological terms, including prevalence, mean intensity and abundance, were applied as defined by Bush et al. (1997). For all length, width and weight measurements we calculated the mean and SD. All statistical calculations and tests were performed in GraphPadPrism (Version 10.4.1, Boston MA, USA).

## 3 Results

### 3.1 Seals examined

The mean weight and length of 13 harbour seals were 30.73 kg (range 25.10–34.30 kg, SD 3.18) and 110.08 cm (range 88.00–135.20 cm, SD16.07), respectively. In addition, the mean weight of the stomach and intestine were 0.37 kg (0.27–0.48 kg, SD 67.01) and 1.09 kg (0.68–1.43 kg, SD 0.22 kg), respectively.

### 3.2 Infection of seals

In the thirteen seals we recovered 4925 nematodes, 790 acanthocephalans, and three cestodes (Supplementary Table S2) (Fig. 1). The prevalences of the nematodes, acanthocephalans and cestodes were 100 %, 100 % and 7.69 %, respectively. The mean intensity of infection for the nematodes (all three genera taken together) was 378.0 (range 4-1790; SD 476). The mean intensity for acanthocephalans was 60.8 (range 4–222; SD 61) and for cestodes it was 3 (only one seal infected with three cestodes) (Table 1).

**Fig. 1:**
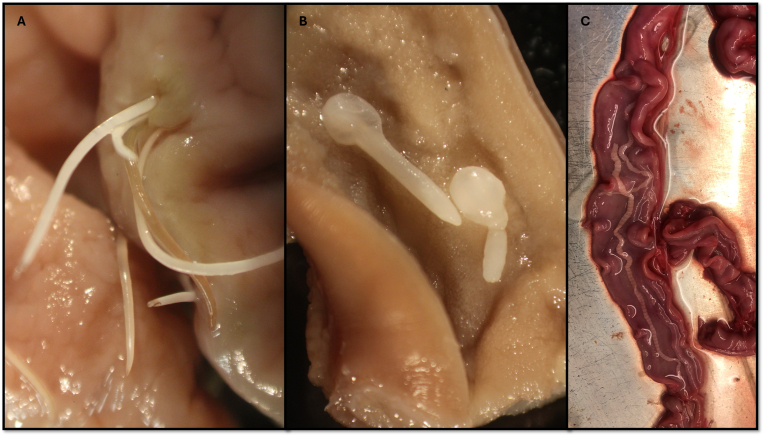
Parasitic helminths attached to the gastrointestinal tract of harbour seal from Limfjorden, Denmark. A. Anisakid nematodes penetrating the stomach mucosa. B. Acanthocephalan *Corynosoma strumosum* attached to the intestinal mucosa of harbour seal. C. Cestode *Dibothriocephalus schistochilus* attached to the intestinal mucosa of harbour seal.

**Table 1:** Prevalence (%), intensity of infection (mean and range) and abundance of anisakid nematodes, acanthocephalans and cestodes in harbour seal *Phoca vitulina* from Limfjorden (Denmark). ∗: only one seal was infected with cestodes.

Parasite genus level	Prevalence (%)	Mean intensity	Abundance	Range	SD
*Anisakis*	84.61	156.1	132.1	1–1560	466
*Phocanema*	100	84.9	84.9	2–269	92
*Contracaecum*	84.61	191.3	161.9	5–440	153
*Corynosoma*	100	60.8	60.8	4–222	61
*Dibothriocephalus*	7.69	3	0.2	3	NA∗

#### 3.2.1 Genus-level morphological identification

Morphological examination by microscopy (Fig. 2) of the 4925 nematodes resulted in assignments of 2104 specimens to the genera *Contracaecum*, 1717 to *Anisakis* and 1104 to *Phocanema* (Supplementary Table S2). The assignment of the cestodes to the genus *Dibothriocephalus* was based on the scolex morphology (presence of two bothria, absence of sclerites). The acanthocephalans were assigned to the genus *Corynosoma* based on the proboscis morphology and tegumental spine patterns (absence of spines on the middle part of the body). For species determination, assignment of the worms to *C. strumosum*, male worm body size and morphology of the testes and cement glands was used as well.

**Fig. 2:**
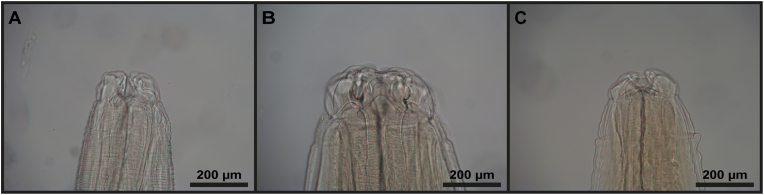
Anterior ends of anisakid worms showing characteristic morphology of labiae of the worms isolated from harbour seal. A. *Anisakis simplex. B. Contracaecum osculatum. C. Phocanema decipiens*.

#### 3.2.2 Species-level molecular identification

Following morphological identification to the genus level we further identified the parasites to species level using molecular methods. Phylogenetic analyses of *cox2* mitochondrial DNA (mtDNA) (Supplementary File S3) and ITS nuclear DNA (nucDNA) (Supplementary File S4) of the nematodes resulted in assignment to *A. simplex*, *C. osculatum*, *P. decipens* and *P. krabbei*. Acanthocephalans and cestodes were similarly identified as *Corynosoma strumosum* and *Dibothriocephalus schistochilus*, respectively (Supplementary Table S1).

With regard to identification of species within the family Anisakidae, 118 nematodes were subjected to PCR targeting the nuclear full-length ITS region and the mitochondrial *cox2* gene. All primer combinations described in Supplementary File S1 were used and a schematic overview can be found in S1 in the sheet named “Identity analysis”. In the ITS sequence, 11 variants were identified in 118 nematodes. For *cox2*, 104 variants were identified in 115 nematodes. These variants were subjected to phylogenetic analysis resulting in two phylograms shown in Supplementary Files S3 (*cox2*) and S4 (ITS). Based on the analysis of *cox2*, 27 specimens grouped closely with *A. simplex* (*s.s.*), 47 with *C. osculatum* (*s.s.*), 16 with *P. decipiens* and 21 with *P. krabbei*. For the ITS region, 29 specimens were assigned to *A. simplex* (*s.s.*), 51 to *C. osculatum* (*s.s.*), 18 to *P. decipiens* and 20 to *P. krabbei*. When the individual nematodes were assigned to the same species by both *cox2* and ITS analyses we recorded the species accordingly.

*Dibothriocephalus schistochilus* was molecularly identified using sequences of two overlapping PCR products amplified using the primers ERIB1 and ERIB 10 for 18S rRNA (ribosomal RNA) and the primers PDG_18S_F5 and BD2 for 18S rRNA to 28S rRNA (Supplementary File S1). Three specimens were sequenced, and three identical 3366 bp long sequences (spanning from the start of 18S rRNA to the start of the 28S rRNA) were obtained. This molecular identification was supported by the morphological analysis. This product showed 100 % identity towards the GenBank acc.no. MW601833 upon NCBI BLAST search.

A total of 24 of acanthocephalan specimens were subjected to PCR using the primer set PDG_18S_F5 and NLR1270. This resulted in 2644 bp long products. Two of the products were completely sequenced and found to consist of 18S rRNA (478 bp), ITS1 (321 bp), 5.8S rRNA (156 bp), ITS2 (248 bp) and 28S rRNA (1393 bp). NCBI BLAST revealed a fragmented result as no entry in GenBank covered the complete sequence obtained in this study. These fragments covered three parts with minor overlaps: 3′ end of 18sRNA, ITS1 + 3′ end of 5.8rRNA, and 5′ end of 5.8S rRNA + ITS2 + 5’ end of 28S rRNA. NCBI BLAST of the different parts revealed several *Corynosoma* species, with high identities (98 %–100 %). However, high percent identities were in general towards *C. strumosum* (e.g. 18S rRNA 100 % and 28S rRNA 99.71 %). The remaining 22 specimens were partly sequenced. Molecular delimitation of the *Corynosoma* species was less clear, so the designation of our samples to the species *C. strumosum* was supported primarily by the morphological and morphometric analysis, which the characteristic proboscis hook shape, number of hook rows and distribution of tegumental spines.

### 3.3 Egg dimensions

**Fig. 3:**
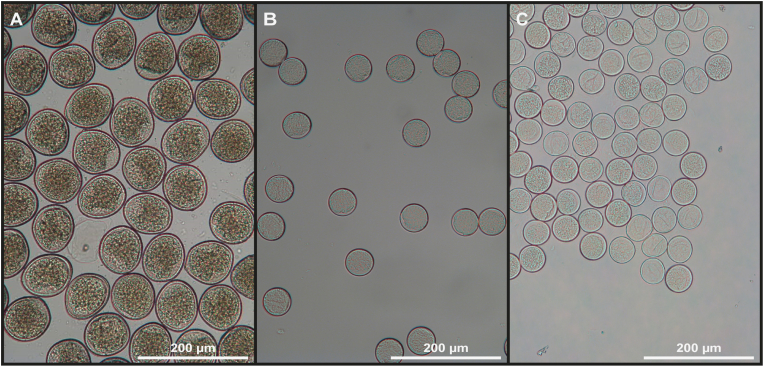
Nematode egg morphology. A. *Contracaecum osculatum*. B. *Anisakis simplex.* C. *Phocanema decipiens*.

The shapes of mature eggs recovered from sexually mature female nematodes were spherical to subspherical and their lengths and widths were recorded (ten eggs per worm measured) (Fig. 3).

The mean egg size of eggs (all egg measurements combined from all eggs isolated from adult female worms) of different species of anisakids were: length 49.1 μm (SD ± 1.9) and width 48.9 μm (SD ± 2.1) for *A. simplex*, length 83.6 μm (SD ± 4.1) and width 83.3 μm (SD ± 3.7) for *C. osculatum*, length 47.8 μm (SD ± 2.6) and width 47.2 μm (SD ± 2.6) for *P. decipiens* and length 47.5 μm (SD ± 2.6) and width 47.5 μm (SD ± 2.6) for *P. krabbei* (see Table 2 for the measurements of eggs from the individual female worms).

**Table 2:** Total number of eggs per female worm (TNEPW) and their size (ten mature eggs per worm measured) from individual female nematodes in harbour seal. Parasites assigned to genus and species level (identified by molecular and morphological methods). GenBank accession nos. for the isolates are shown in Supplementary Table S1. ∗: eggs lost during processing and thereby not size measured.

Anisakis simplex				Contracaecum osculatum				*Phocanema decipens,(PD)* Phocanema krabbei (PK)			
Sample ID	TNEPW	Mean Length + SD	Mean Width + SD	Sample ID	TNEPW	Mean Length + SD	Mean Width + SD	Sample ID	TNEPW	Mean Length + SD	Mean Width + SD
PV03_N23e	30,170	49.5 μm *± 1.6*	48.5 μm *± 2.4*	PV03_ N22e	12,432	84.5 μm *± 1.6*	83.5 μm *± 2.4*	PV03_ 24e (PD)	21,440	NA∗	NA∗
PV03_N25e	72,810	46.5 μm *± 2.4*	46.5 μm *± 2.4*	PV05_ N18e	9751	83.0 μm *± 2.6*	84.5 μm *± 2.8*	PV05_N16e (PD)	10,339	47.8 μm *± 2.6*	47.2 μm *± 2.6*
PV03_N27e	20,773	49.5 μm *± 1.6*	49.5 μm *± 1.6*	PV06_ N10e	25,337	85.5 μm *± 3.7*	84.0 μm *± 3.9*	PV11_N19e (PK)	9818	48.9 μm *± 2.2*	48.9 μm *± 2.2*
PV03_N28e	25,954	50 μm *± 0.0*	50 μm *± 0.0*	PV06_ N11e	39,248	83.5 μm *± 4.1*	81.5 μm *± 2.4*	PV12_N13e (PK)	8787	46.2 μm *± 2.2*	46.1 μm *± 2.2*
PV03_N29e	7202	50 μm *± 0.0*	50 μm *± 0.0*	PV06_ N12e	16,341	80.0 μm *± 3.3*	79.0 μm *± 3.9*				
				PV06_ N13e	20,055	80 μm *± 2.4*	80 μm *± 2.4*				
				PV12_ N10e	19,464	85.5 μm *± 2.8*	84.0 μm *± 3.2*				
				PV12_ N11e	7449	88.0 μm *± 3.5*	88.0 μm *± 3.5*				
				PV12_ N12e	9156	84.0 μm *± 3.2*	83.5 μm *± 2.4*				

### 3.4 Egg counts in adult female nematodes

We directly counted the number of mature eggs for each nematode species. *A. simplex* had a mean of 31,382 eggs (min: 7202; max: 72,810; SD: 24,721); *C. osculatum* had a mean of 17,693 (min: 7449; max: 39,248; SD: 10,004); *P. decipiens* had a mean of 15,890 (min: 10,339; max: 21,440; SD: 7850); and *P. krabbei* had a mean of 9303 (min: 8787; max: 9818; SD: 729). To provide a general estimate of the numbers of nematode eggs in adult female anisakid worms we combined data for all species and calculated the mean number of anisakid eggs per worm as 20,363 (Table 2).

#### 3.4.1 Estimated parasite egg release and infection pressure

Based on the average number of mature eggs from adult females of the four nematode species, we estimated the potential release of nematode eggs from a harbour seal. The most heavily nematode infected seal (PV03) carried 1790 nematodes, of which 463 had reached the adult stage (both male and female worms). We aimed to determine a ratio of how many adult female worms could be found in a seal in relation to the total number of worms (males and females, mature and immature) from the same seal. As we recorded mature eggs in uteri of 36 out of 140 randomly selected female nematodes we applied a ratio of 36/140. This ratio was then used to estimate the number of mature female nematodes in the seal. Accordingly, we estimated that a seal carrying 463 adult nematodes would have 119 adult female nematodes with mature eggs in the stomach. When applying the calculated mean number of eggs in a worm uterus (20,363 eggs) (see 3.4), we then estimated the egg release from this heavily infected seal to reach at least 2,423,197 (20,363 × 119) eggs. Provided that all mature eggs were released within one day (not yet documented) this would suggest a daily parasite egg release per seal of approximately 2.5 × 10^6^ eggs. However, the infection intensity varied between 5 and 440 (mean: 191; SD 153) parasites per host for *Contracaecum*, between 2 and 269 (mean: 85; SD 92) for *Phocanema* and between 1 and 1560 (mean: 156; SD 466) for *Anisakis*. The mean number of adult anisakid nematodes in the examined seals was 68 (SD 124) and 17.5 (68 × 36/140) of these are considered egg producing, which leads to a mean output from a seal of 356,353 (20,363 × 17.5) eggs per day.

## 4 Discussion

Populations of harbour seal *Phoca vitulina* have increased markedly in the Baltic Sea and adjacent inner Danish waters over the last 20 years Harding et al., 2023). Various species of pinnipeds, including harbour seal, are known as hosts to a range of parasites (Borgsteede et al., 1991; Aspholm et al., 1995; Marcogliese et al., 1996; Ólafsdóttir and Hauksson, 1998; Stobo et al., 2002; Szostakowska et al., 2002; Skrzypczak et al., 2014). The occurrence of anisakid nematodes in harbour seals in these earlier studies varied from relatively low prevalences of 58 % (Borgsteede et al., 1991) and 66 % in the North Sea (Lehnert et al., 2007) up to 100 % in the North Atlantic (Ólafsdóttir and Hauksson, 1998). We here show that the harbour seal population in Danish waters also carries significant infections with 100 % prevalences for nematodes and acanthocephalans. Some of the nematode species found in Danish seals were previously reported in other seal habitat (Lunneryd, 1991), but our finding of *P. krabbei* in the present study is a new record. In addition, the cestode *D. schistochilus* was not reported in the area previously.

The gastrointestinal anisakids of seals use invertebrate (e.g. crustaceans) and vertebrate (fish) hosts in their life cycles (Fagerholm, 1982; Køie et al., 1995; Køie and Fagerholm, 1995; Zuo et al., 2018; Nadolna-Altyn et al., 2017; Pawlak et al., 2019). Anisakid larvae in fish (paratenic host) may have an impact on their health, growth and quality, which was suggested by several studies on commercial species (Hafsteinsson and Rizvi, 1987; McClelland, 2002; Ryberg et al., 2020, 2021; Øvegård et al., 2022). It is therefore noteworthy that various anisakid nematode species are prevalent in fish populations in inner Danish waters and adjacent regions (Perdiguero-Alonso et al., 2008; Buchmann and Kania, 2012; Mehrdana et al., 2014; Sokolova et al., 2018; Severin et al., 2020; Karami et al., 2022; Kumas et al., 2024). Some North Sea herring populations may perform seasonal migration towards the Baltic Sea (Grabda, 1974, 1976), which suggests some exchange between anisakid parasite populations in the Atlantic (Jensen et al., 1994; Gay et al., 2018) and the inner Danish waters. This study documents that there is a link between seals in the marine habitat and infections in fish. The North Sea harbour seals and their parasitic infections were reported during the 1990s (Lehnert et al., 2007), and we here present data documenting an infection prevalence of 100 % with anisakid nematodes in inner Danish waters. However, in order to assess the infection pressure and impact of nematode parasites on the local fish populations, we need to measure the fecundity (production of eggs) of the nematode parasites found in the seals.

Previous studies on the egg content in these nematode species were based on either *in vitro* culture of worms (Moratal et al., 2023) or indirect measurement of eggs *in situ* in worm uteri (Herreras et al., 2007). The present study determined the infection level in seals from the Danish marine area Limfjorden and secondarily estimated the reproductive output of nematodes parasitizing the seals. This was done by isolating and counting all proteinase resistant-eggs from the adult female nematodes. First, the prevalence of infection for all four anisakid nematodes was 100 %, therefore allowing us to provide tentative data for the infection pressure on fish exerted by the seal population in specified areas. All four nematode species were represented by both juvenile and adult forms. However, based on the recorded ratio between total number of worms and mature egg-producing worms it could be calculated that more than 2 × 10^6^ mature eggs are present in the anisakid nematodes in at least one heavily infected seal (based on the maximum worm burden in this study). When considering all seals examined, we estimate a mean number of more than 300,000 eggs are present in each seals. Classical studies on oviposition rates of other ascarids, such as *Ascaris suum* in pigs, suggest far higher egg release rates per day (Kelley and Smith, 1956; Olsen et al., 1958). These investigations were based on egg flotation methods applied for pig faeces, and subsequent examination of pigs for adult female worms. It was shown that more than 1 × 10^6^ eggs were released by one female worm per day. Our seal data suggested a lower daily egg output, but it should be emphasized that the anisakid worms in question are far smaller than *A. suum*. As size of the parasites is likely to affect fecundity, we hypothesize that the size difference is the reason for this recorded difference of the total egg output per female worm. No controlled *in vivo* data on the daily output from female anisakid worms are available, but *in vitro* studies (Moratal et al., 2023) suggest that the daily output per individual female *Anisakis* worm is near 30,000 eggs per day. This corresponds very well with the mature egg content of one female *A. simplex* in our study, and therefore, it cannot be excluded that the egg loads (directly isolated and counted in this study) in female worms are released within one day. This should be confirmed in future experimental settings. However, all mature eggs have the potential to hatch, release an infective larva and infect a paratenic host (Køie et al., 1995), and each thus contributes to the overall infection risk. In this study we did not estimate the release of parasite eggs from the acanthocephalans and the cestodes, because compared to the nematode levels the infection intensities in the seals were considerably lower. However, future analyses related to these groups should be conducted as well.

Apart from eliciting pathological reactions in fish it should be noted that anisakid parasites are potentially zoonotic (Mercado et al., 2001; Torres et al., 2007; Strøm et al., 2015; Murata et al., 2021; EFSA BIOHAZ Panel, I et al., 2024). Human cases have been reported from the regions around the inner Danish waters for *A. simplex* (Andreassen and Jørring, 1970), *C. osculatum* (Schaum and Müller, 1967) and *P. decipiens* (Nordholm et al., 2020; Mejer et al., 2022). A similar concern with regard to fish parasites of public health relevance exists for cestodes (Schaeffner et al., 2018) and acanthocephalans in seals (Buchmann and Karami, 2024). Acanthocephalans within the genus *Corynosoma* occur in local fish populations (Setyawan et al., 2020). Severe human cases caused by *C. validum* and *C. villosum* were reported from Japan (Takahasi et al., 2016; Fujita et al., 2016), which may be explained by the infection prevalences of commercial fish species in the marine areas around Japan (Sasaki et al., 2019). The acanthocephalan *C. strumosum,* recorded in the present study, has been recovered from human patients (Schmidt, 1971) emphasizing the potential infectivity of this species for humans. Two species of the cestode genus *Dibothriocephalus* (*D. latus*, *D. dendriticus*) are considered infective to humans (Schaeffner et al., 2018), and *D. schistochilus* may be able to infect humans as well. The closely related *D. lanceolatum* has been reported to infect both dogs and humans (Rausch and Hilliard, 1970), although no confirmed human *D. schistochilus* cases are available in the literature at present. Controlled infection studies involving this cestode species are therefore recommended.

North Sea and Atlantic marine area studies have documented the unequivocal connection between seal occurrence and infection of fish with *P. decipiens* (McClelland and Martell, 2001; Hauksson, 2002; Jensen and Idås, 1992; Kuhn et al., 2013). The information on occurrence of parasites in harbour seals in the inner Danish waters rely on older studies based on seals found dead during a canine distemper virus epidemic in 1988 (Lunneryd, 1991). The present updated study on harbour seals in the Danish seas may provide a basis for a future assessment of seal impacts on fish in Danish waters. The study may also remodel our views on links in the ecosystems. Although representatives of the genus *Anisakis* carry the vernacular name “whale worms”, as they reach their sexually mature stage in whales, we showed that *A. simplex* also produces significant, reproductive populations in at least one species of pinnipeds, the harbour seal. This finding must be included in future models estimating impact of seals on fish populations. The harbour seal population in waters connected to the inner Danish marine areas has been estimated at 9000–11,000 individuals in Kattegat, and at 5000 individuals in Skagerrak (Harding et al., 2023). The total number of mature parasite eggs present (and possibly the number released on a daily basis) in the harbour seals may therefore exceed 10 × 10^9^. On an annual basis the release of eggs would thus be considerable, potentially resulting in a very high risk of infection to fish consuming the first paratenic hosts (crustaceans). In this context it should be noted that a range of commercial fish species in the inner Danish waters are considered target species including cod, whiting, plaice, flounder, dab, turbot and even pelagic species such as clupeids. The direct and indirect effects of anisakid nematode larvae on fish health should be elucidated in future studies. This would allow fish ecology models to consider parasitism, along with other biotic and abiotic factors, in future studies. Thereby, surveys of parasite infections in seals may be applicable not only for the economic impact on the fish processing industry, due to increased occurrence of anisakids in fish products, but may even contribute to improved management of fish stocks.

## CRediT authorship contribution statement

**Kaan Kumas:** Writing – review & editing, Visualization, Validation, Methodology, Investigation, Formal analysis, Conceptualization. **Carlota Marola Fernandez Gonzalez:** Writing – review & editing, Methodology, Investigation. **Per Walter Kania:** Writing – review & editing, Validation, Investigation, Formal analysis. **Kurt Buchmann:** Writing – review & editing, Writing – original draft, Validation, Project administration, Methodology, Investigation, Funding acquisition, Formal analysis, Conceptualization.

## Authorship confirmation form

All authors have participated in a) conception and design, or analysis and interpretation of data; b) drafting the manuscript or revising it critically and c) approval of the final version.

## Funding

The present study was supported by the European Fisheries and Marine Fund by the grant to the SEAL project (EFMVB-230034) and the 10.13039/501100022592GUDP funded project OPTIKVAL (Ministry of Food, Agriculture and Fisheries grant 34009-22-2100).

## Declaration of competing interest

The authors declare that they have no conflict of interests.

## Figures and Tables

**figs1:**
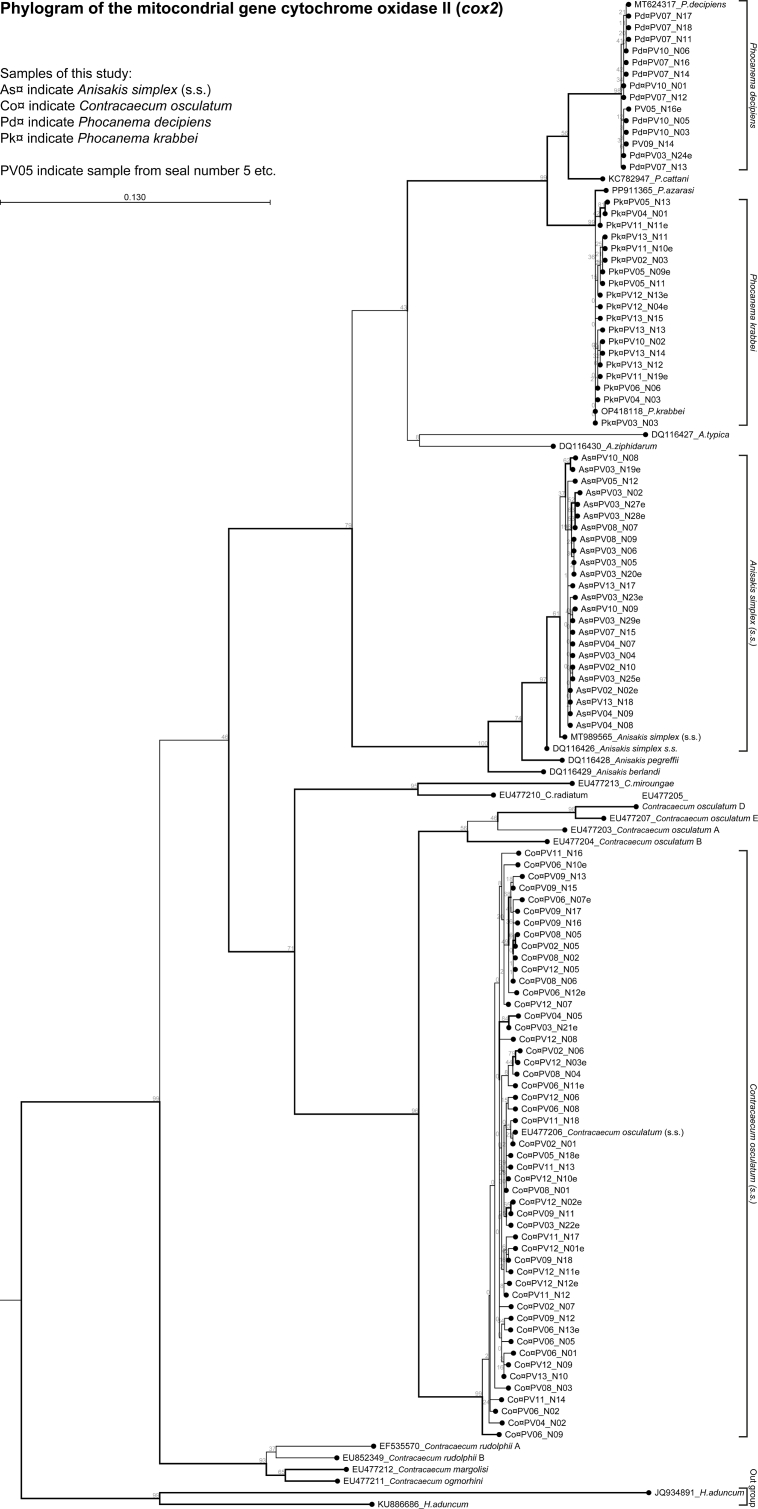


**figs2:**
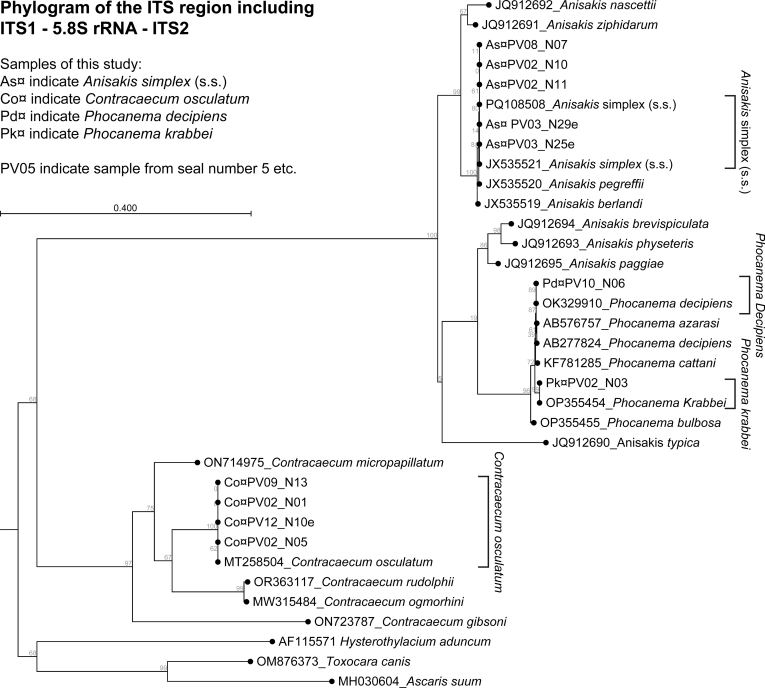


## Appendix A Supplementary data

Note:Supplementary files (S1 and S2) are associated with this article.
