# Supplementary material for: Gastrointestinal parasites of harbour seal (Phoca vitulina L.) in Danish marine waters: Prevalence, abundance, intensity and reproductive potential
Source: Int J Parasitol Parasites Wildl. 2025 Apr 1;27:101066. doi: 10.1016/j.ijppaw.2025.101066 (PMC12002897; doi:10.1016/j.ijppaw.2025.101066)
Supplement: Multimedia component 2 [file mmc2.docx]

**Supplementary Table 2.**

1. **The number of parasites assigned to genus level in the 13 individual harbour seals**

| Seal No | *Phocanema* | *Anisakis* | *Contracaecum* | *Corynosoma* | *Dibothriocephalus* |
| --- | --- | --- | --- | --- | --- |
| PV01 | 3 | 1 | 0 | 4 | 0 |
| PV02 | 26 | 27 | 65 | 18 | 0 |
| PV03 | 153 | 1560 | 77 | 88 | 3 |
| PV04 | 103 | 68 | 236 | 33 | 0 |
| PV05 | 166 | 6 | 149 | 33 | 0 |
| PV06 | 40 | 13 | 440 | 222 | 0 |
| PV07 | 10 | 1 | 0 | 4 | 0 |
| PV08 | 2 | 6 | 37 | 19 | 0 |
| PV09 | 16 | 2 | 149 | 76 | 0 |
| PV10 | 20 | 7 | 5 | 24 | 0 |
| PV11 | 237 | 0 | 421 | 121 | 0 |
| PV12 | 61 | 0 | 350 | 60 | 0 |
| PV13 | 269 | 26 | 176 | 88 | 0 |

1. **Proportions of the different nematode genera in individual seals. Nematodes in harbour seals identified using morphological and molecular methods.**

|  |  | Identified subsamples | | | Percentage occurrence of different genera in individual seals | | |
| --- | --- | --- | --- | --- | --- | --- | --- |
| Seal No | Total no. Examined Nematodes | *Phocanema* | *Anisakis* | *Contracaecum* | *Phocanema* | *Anisakis* | *Contracaecum* |
| PV01 | 4 | 3 | 1 | 0 | 75% | 25% | 0% |
| PV02 | 105 | 23 | 24 | 58 | 21.9048% | 22.8571% | 55.2381% |
| PV03 | 140 | 12 | 122 | 6 | 8.5714% | 87.1429% | 4.2857% |
| PV04 | 107 | 27 | 18 | 62 | 25.2336% | 16.8224% | 57.9439% |
| PV05 | 112 | 58 | 2 | 52 | 51.7857% | 1.7857% | 46.4286% |
| PV06 | 111 | 9 | 3 | 99 | 8.1081% | 2.7027% | 89.1892% |
| PV07 | 11 | 10 | 1 | 0 | 90.9091% | 9.0909% | 0% |
| PV08 | 45 | 2 | 6 | 37 | 4.4444% | 13.3333% | 82.2222% |
| PV09 | 107 | 10 | 1 | 96 | 9.3458% | 0.9346% | 89.7196% |
| PV10 | 32 | 20 | 7 | 5 | 62.5% | 21.875% | 15.625% |
| PV11 | 122 | 44 | 0 | 78 | 36.0656% | 0% | 63.9344% |
| PV12 | 129 | 19 | 0 | 110 | 14.7287% | 0% | 85.2713% |
| PV13 | 107 | 61 | 6 | 40 | 57.0093% | 5.6075% | 37.3832% |
